# Patient Perceptions of a Digitally Enabled Community Health Worker Intervention: Qualitative Study Among Pilot Trial Participants

**DOI:** 10.2196/93288

**Published:** 2026-06-04

**Authors:** Jocelyn Carter, Natalia Swack, Narmeen Rehman, Yadira Reyes-Richards, Karen Donelan, Anne Thorndike

**Affiliations:** 1 Division of General Internal Medicine, Massachusetts General Hospital, 55 Fruit Street, Blake 15, Boston, MA, United States, 1 617-726-9000; 2 Department of Internal Medicine, Henry Ford Hospital, Detroit, MI, United States; 3 Department of Medicine, Brandeis University, 415 South Street, Waltham, MA, United States

**Keywords:** heart failure, digital platform, remote monitoring, home-based care, community health worker, social needs care

## Abstract

**Background:**

Most studies assessing digital interventions for people with heart failure (HF) focus on clinical outcomes, and few include patient perspectives. Understanding patient experiences of the use of a digital HF platform along with community health worker (CHW) care as part of a digitally enabled CHW intervention can inform management of HF at home and improve the postdischarge phase of care.

**Objective:**

This study aimed to identify patient perceptions related to the use of a digitally enabled CHW intervention.

**Methods:**

This qualitative study included interviews with adults (aged ≥18 years) with HF who were assigned to the intervention arm of a pilot randomized controlled trial from September 2022 to June 2023. For 30 days after hospital discharge, intervention participants were paired with a CHW and instructed to use a digital platform that tracked biometrics (eg, heart rate, oxygenation, blood pressure, body weight, steps taken, and symptoms) and offered educational videos. In-depth interviews were conducted after the 30-day intervention was complete (between 31 and 45 days after hospital discharge). Key interview domains included barriers and facilitators to the intervention, use of remote monitoring in HF, and the role of CHWs in HF home care.

**Results:**

Interviews with participants (N=19; mean age 62.1, SD 15.1 years) yielded five key themes: (1) the combined intervention was well received, and CHWs made the use of the digital platform more approachable; (2) the digital platform enhanced HF knowledge and confidence in self-care; (3) digital platform use was easy to integrate into daily routines; (4) in addition to assisting with navigation of unmet social needs (eg, transportation, insurance benefits, and food access), CHWs provided emotional support and increased motivation for clinical care plan adherence and platform use; and (5) connectivity issues and other technical challenges occurred with digital platform use.

**Conclusions:**

The digital platform was easily integrated into patients’ daily routines. CHWs played a key role in making the platform more approachable for participant use. Further research is needed to better understand the impact of this intervention in larger HF populations over more extended time intervals.

## Introduction

Heart failure (HF) remains a leading cause of hospitalizations and readmissions in the United States and contributes to nearly 6.5 million inpatient hospital stays each year [1-3]. Despite policy changes, comprehensive case management strategies, and breakthroughs in pharmacological therapy [4-6], HF-related hospitalizations and their expenditures continue to rise [7]. This is, in part, driven by patients managing HF at home who often face numerous clinical (eg, tight dietary restrictions, rigid medication regimens, and daily symptom monitoring) and social (eg, transportation, food security, housing stability, and access to care) challenges [8-10]. These barriers are associated with worsening HF morbidity, mortality, and adverse outcomes [11,12]. Ultimately, these conditions can lead to HF exacerbations, increased use of urgent or emergent care, and missed opportunities for primary and preventive care after hospital discharge [13].

Digital interventions, particularly in the postdischarge setting, have emerged as a promising tool to improve HF clinical outcomes [14,15]. Digital platforms that closely monitor vital signs, activity, and symptom data have helped improve the value of remote monitoring in managing HF at home [15,16]. Most digital studies in HF are focused on usability or efficacy relevant to clinical outcomes such as reducing hospitalizations and emergency department visits [15]. Few clinical trials assessing digital interventions in HF evaluate patient perspectives on their impressions of the digital intervention itself. Understanding the patient experience with digital interventions can inform key barriers and facilitators to managing HF care at home [17,18].

In a pilot randomized controlled trial [19], a digitally enabled community health worker (CHW) intervention for patients with HF demonstrated intervention feasibility and improvements in managing HF at home. In the full sample (control: n=31; intervention: n=25), fewer participants in the intervention group were readmitted 30 days after hospital discharge compared to the control group (n=3, 12% vs n=8, 26%; *P*=.12). Both arms had similar rates of missed clinic appointments and emergency department visits. To understand the patient experience with combining a digital platform with social support from a CHW [20], qualitative interviews were performed with intervention participants, who were paired with a CHW and instructed to use a digital platform. Our study aim was to better understand the barriers and facilitators associated with use of the digitally enabled CHW intervention, CHW home care, remote monitoring, and perceptions of digital health tools in a HF population.

## Methods

### Setting and Study Design

This was a qualitative study of participants who received the 30-day digitally enabled CHW intervention in a pilot randomized controlled trial. All participants had a diagnosis of HF listed on their problem list within the electronic medical record, were hospitalized at the time of enrollment, and had been hospitalized at least once in the 12 months prior to enrollment. Participants were initially identified for trial participation through inpatient electronic health records at an academic medical center. Intervention participants were paired with a CHW and the digital platform. The digital platform was connected to a mobile phone app with features including a digital weight scale, a digital blood pressure monitor, a symptom questionnaire, educational videos, and an arm-worn sensor monitoring oxygenation, steps taken daily, and heart rate. The pilot trial details, including procedural flow, inclusion criteria, and study design, are described in a previous publication [19]. Of the 19 participants who completed the intervention, 19 were successfully contacted for qualitative interviews.

### Interview Protocol and Measures

All interviews were conducted after the intervention via telephone 31 to 45 days after hospital discharge, and interviews lasted 20 to 45 minutes in length. An interview guide was developed through key informant interviews with patients, HF specialists, qualitative research experts, senior scientists, health services researchers, and primary care physicians. This was coupled with a review of literature associated with prior qualitative HF studies [21]. The guide covered prespecified domains, and consistency was ensured by having all interviews conducted by a senior clinical researcher (NS). None of the researchers or coauthors in this study were involved in direct patient clinical care or the delivery of the trial intervention. Pretesting of the interview guide was performed with 3 participants; no additional modifications were made and the 3 pretesting participants were included in the full sample. The interview guide included 21 items in total, and major domains were barriers and facilitators to managing HF care at home, use of CHW care in HF at home, remote monitoring in HF, and perceptions related to use of technology in HF (Multimedia Appendix 1). All procedures were approved by the Mass General Brigham Institutional Review Board (protocol number 2018P002014) on September 9, 2022, and all participants provided written informed consent.

### Analysis

Transcripts from interviews were analyzed verbatim with the Dedoose (version 8.3.47b) platform. An analytic framework was developed based on the recurring themes that emerged during interviews. To optimize reliability, 2 members of the study team (NS and JC) independently reviewed the data, identified themes, and collaboratively applied the framework to categorize and interpret findings. Weekly meetings occurred for discussions focused on data interpretation and common themes. Content analysis was completed by each coder for each patient interview prior to meeting; this facilitated consensus for all generated codes. Intercoder reliability was achieved and any discrepancies were resolved with a third researcher (KD) with expertise in qualitative research and patient interviews. The available cohort was exhausted and all responses generated by interview participant responses were analyzed. Structured chart reviews were also performed via the electronic health record and stored in a Research Electronic Data Capture (REDCap; Vanderbilt University) database [22]. Data related to demographic information, insurance status, education, and major medical and psychiatric comorbidities were collected in the chart review.

### Rigor

The research team used a formal report generated by the electronic medical record to identify eligible patients. Screening for eligibility was completed by research interns trained to assess eligibility, approach, and enroll participants as a part of standardized research procedures. Participants were not randomized to the intervention or control arm until all standardized study enrollment procedures were complete. Research interns were unaware of the randomization assignments until after all enrollment procedures were complete. In addition, the qualitative interview guide was developed to standardize questions asked of participants, probe any anticipated answers, and accurately capture the participant perspective. The research team was also mindful of the extent to which data can change over time during analysis and tracked all coding decisions meticulously. While the perspectives gathered from participants may not apply to those in other or non-HF populations, the themes may apply to other cohorts with similar characteristics. The small sample size facilitated in-depth and authentic interviews reflective of the patient experience. While the nature of qualitative studies can make replication challenging, the research focus was on gathering individual perspectives to add to the evidence base for HF care at home.

### Ethical Considerations

Institutional review board approval was obtained from the Mass General Brigham Human Research Committee on September 9, 2022 (2018P002014). All enrolled participants provided written informed consent prior to this study. Data were anonymized before analysis to ensure confidentiality. All study data reported were deidentified. For remuneration, US $50 was provided to participants at the time of enrollment and an additional US $200 was provided after successful study completion. All methods were carried out in accordance with guidelines and regulations outlined by the Mass General Brigham Institutional Review Board.

## Results

Of the 19 pilot trial intervention participants, 19 were interviewed (Multimedia Appendix 2) after completing the 30-day digitally enabled CHW intervention. Participant characteristics included a mean age of 62.1 (SD 15.1) years, with most participants identifying as male (n=10, 53%), White (n=11, 58%), and used Medicare (n=8, 42%) or commercial insurance (n=9, 47%). Thematic analysis identified 5 key themes, highlighting how participants experienced the integrated digital platform and CHW support. These themes are described below.

### Theme 1: The Combined Intervention Was Well Received, and CHWs Made the Use of the Digital Platform More Approachable

One of the most common themes described by participants was how well the intervention worked in terms of helping them manage their HF at home. Several individuals emphasized that the platform and CHW outreach complemented one another by helping reduce anxiety about self- management and facilitate engagement in care. One participant noted the following:

*I never felt overwhelmed by the platform or working with the CHW. Just the opposite. I felt that both eased a lot of anxieties I have and helped me to filter my way through the maze.*

Participants also noted that CHW staff played a key role in making the digital platform more approachable, especially for those unfamiliar with the technology. This was particularly true for those that had not used digital platforms in the past.

### Theme 2: The Digital Platform Enhanced HF Knowledge and Confidence in Self-Care

Participants reported that the intervention helped them better understand their HF and how their self-care behaviors directly impacted their health. Several individuals described gaining new knowledge about the importance of tracking vital signs, medication adherence, and recognizing early signs of exacerbation. As one participant noted,

*This was really helpful to have the blood pressure and weight and a person that you could stay on track with. Otherwise, sometimes you could end up in the ED for something small if you didn’t have clear information or hear back from your PCP quickly enough.*

Others described how education from the intervention and real-time feedback from the monitoring tools prompted changes in their behavior, including more consistent medication use and routine self-monitoring.

### Theme 3: Digital Platform Use Was Easy to Integrate Into Daily Routines

Participants reported that the digital health platform fit easily into their daily routines and supported consistent engagement with self-care activities. Several individuals described the process of using the device as quick and manageable, with one noting,

*My daily sessions with the technology were less than 10 minutes in the morning to do the blood pressure and weight which was great.*

Others emphasized how the platform helped reinforce new habits for routine monitoring that they had previously struggled to adopt, despite prior clinical advice. A number of participants shared that the consistency of daily use also appeared to support habit formation over time.

### Theme 4: In Addition to CHWs Assisting With Navigation of Social Barriers to Care, CHWs Provided Emotional Support and Motivation for Clinical Care Plan Adherence

Participants described CHW care as a consistent and accessible source of support during the intervention. Participants offered that CHWs assisted with helping clarify their care plans, providing accountability for managing their HF, and supporting use of the digital platform. One participant shared their CHW helped them stay on track through regular check-ins:

*I did like having a person that I could speak with to keep me motivated - even just once a week or more if needed. I found it very helpful and supportive that there was somebody on the other end.*

A number of participants cited CHW-specific education and health care coaching as an important part of the intervention. Participants also identified CHW staff as a key resource for addressing social needs. Reported areas of support included housing navigation, transportation to appointments, insurance benefit enrollment, and access to food or home health services.

### Theme 5: Connectivity Issues and Other Technical Challenges Occurred With Digital Platform Use

Some participants described specific technical challenges with digital platform use. Most issues related to device connectivity, syncing delays, or sensor functionality. For example, as one participant shared,

*The only problem I had was on occasion the blood pressure cuff did not register a pressure easily. So it took three or four tries before a readable pressure was there, but that was the only thing.*

Other participants had intermittent issues with blood pressure measurement accuracy or precision. Participants expressed preferences to wear the arm sensor while showering for ease of use. As the digital platform’s mobile app was embedded in an Android phone that needed to be carried with participants when leaving their home, some participants expressed interest in having an app that could be used on their personal phone to avoid this. Table 1 summarizes the 5 themes.

**Table 1.** Major themes and illustrative quotes from poststudy interviews with participants with heart failure (HF).

Themes	Theme description	Quotes
Theme 1: the combined intervention was well received, and CHWs^a^ made the use of the digital platform more approachable.	Remote monitoring devices and app-based tools helped patients integrate health monitoring (blood pressure and weight) into daily routines, supporting adherence to care plans and fostering self-awareness of health.	“I didn’t know anything like this existed, so everything was a positive, you know what I mean? And I don’t know, because I’m a little older, but sometimes I have a hard time getting information that I need. And with the app and the CHW, everything was right there. She had the answer to any question I would possibly ask about the app and it made me more willing to use it.” “Sometimes I can have a problem working with new things and new people that I don’t know as well but this was easy. The app was great. She helped me get over any concerns I had about using the technology. Very comfortable and helpful overall.” “People with my condition should want to be in the study and use the devices. It will give them and their family and them a lot more security and comfort knowing that they are being tracked.”
Theme 2: the intervention enhanced HF knowledge and confidence in self-care.	Participants reported gaining a better understanding of their health condition and how self-care at home was connected to their overall health.	“I became more aware of the importance of knowing [the] baseline for my blood pressure and that my weight would fluctuate depending on what time I took my weight. So, I became more consistent with my medication.” “They helped me with a lot of stuff I really didn’t know about and answered questions for me. I mean, it was nice having an advocate.” “I did find a lot of information on my blood pressure. And I’m not a person who takes my blood pressure daily, but it was good information and would frequently remind me, ‘Oh, [expletive], you haven’t had your medication today. What are you doing?’ Because my response to the medication is kind of connected to the blood pressure numbers, so it was eye-opening for me to do that.”
Theme 3: digital platform use was associated with ease of integration into daily routines.	Patients thought that the digital platform was easily integrated into their daily schedules and assisted with reminding them to adhere to clinical care plan details.	“I’ve never been in the habit of weighing myself every day, even though I’ve had a couple of doctors say that I should weigh myself more often, but this technology really changed that.” “It makes it long-lasting in the sense that you’re doing it every day. So, it’s more likely going to become a habit.” “It’s definitely kept me on track. I don’t know that I would’ve gotten into the habit of weighing myself and doing my blood pressure every day if I hadn’t been in this study.” “It makes it very easy to stay on track. And it makes it long-lasting in the sense that you’re doing it every day. So, it’s more likely going to become a habit.”
Theme 4: in addition to CHWs assisting with navigation of social barriers to care, CHWs provided emotional support and motivation for clinical care plan adherence.	Participants felt that CHWs helped address unmet social needs, improved their understanding of how to access needed resources, and provided important social support.	“I can be very hard person to communicate and talk with. I think I know everything. But the CHW, she educated me on the importance of the different medications and how blood pressure and my diet tie into things which was helpful. I never hung up on her or anything, so that was really good [laughter].” “She was able to get the ball rolling with Elder Services so they can come out and do a home evaluation that I had been waiting on for a while.” “When she called, she called frequently, made sure that I understood everything that I needed to understand. And she was a very good person.” “God, I really wish I could keep the CHW forever. [laughter] I’m not going to lie. I just liked the information I was getting and I learned so much- just knowing that someone was supporting me that way was a relief.” “I thought it was great when she assisted me, especially with the Mass Health insurance application and everything.” “The CHW did everything on point. I mean, from helping with the housing and getting help with groceries. She did things that I couldn’t-- I, myself, couldn’t have done and I am the better for it.” “When we were talking about transportation and medical issues, she immediately, sent me an email, letting me know what programs that I qualified for which was great.” I really appreciated having help with getting the fuel assistance program. Getting access to the SNAP program also helped me. The way things are in this world today, just to be able to pay bills is important.” “With the SNAP, she got on the call with me and was able to give them the information that I didn’t know I needed to have in front of me. If I have to call again, I will be ready.”
Theme 5: connectivity issues and other technical challenges occurred with digital platform use.	Participants identified several improvements for the intervention focused on the digital platform.	“I had a couple of times where I weighed myself twice or I did my blood pressure because the thing was still going around a circle after a minute or so or two, and then it didn’t look like it was going to record, so I did it again.” “I found the cuff reading to vary -- it gave me a couple of very high readings that were incorrect and I discussed with CHW. Everything ended up improving after I let you guys know about it.” “It would have been great to hop in the shower and keep the sensor on, but I couldn’t do that because it wasn’t waterproof.” “I had to carry two phones, which it’d be nice if it could work with iOS phone instead of the android.”

a CHW: community health worker.

## Discussion

### Principal Findings

This study demonstrated that digital platform use was feasible and that CHW staff assisted in making the platform more approachable for patients. This work strengthens our understanding of the barriers and facilitators to home-based HF management by identifying patient experiences with a digitally enabled CHW intervention as a part of a pilot trial. Few studies have examined patient experience with digital health interventions in the context of clinical trials. This work deepens the context of patient-centered HF home management identified in our previous studies [18,21] and other prior qualitative studies on HF [23].

Participants indicated that CHW care made the use of the digital platform more acceptable for patients. While CHW care is traditionally applied to social risk navigation [24,25], the use of CHWs specifically in digital trials for HF is novel and represents another way that CHWs can contribute to the landscape of care for patients facing HF at home [26]. Using CHW care along with HF digital platforms may help mitigate barriers to adoption and usability of digital platforms [27-29]. Despite patient hesitation regarding use of digital modalities to manage their health [30], our study findings highlighted positive participant responses regarding use of the digital platform. A recent cross-sectional study of patients with HF found that while only 22% of respondents reported prior use of health-related mobile apps, 53% of respondents expressed interest in using one for future self-management [31]. This suggests that patient interest in use of digital platforms may exceed actual use. Coimplementation of digital strategies with CHW or navigator support, even for a time-limited initiation interval for patients, could be an effective strategy for lowering barriers to digital health adoption among patients with HF.

Participants indicated that routine completion of home-based self-management skills such as monitoring home blood pressure, weight, and taking medications improved with the intervention. This is consistent with other remote monitoring studies in HF and chronic disease [32,33]. While this intervention was only 30 days in length, management skill uptake and integration into daily routines is a critical first step toward reducing preventable readmissions and improving postdischarge care transitions. One national survey of recently hospitalized patients with HF found that those who tracked their weight daily were significantly more likely to seek help for fluid retention than those who did not [34]. A number of small observational studies and trials evaluating mobile apps in HF populations have noted similar findings with improved clinician-patient communication, self-care, and engagement [35,36].

Given the established link between unmet social needs and heightened risk of HF readmission [37,38], our findings underscore the value of CHW care in efforts to help reduce risk associated with urgent and emergent care use. Participants indicated that CHW staff were helpful in assisting with navigating unmet social needs such as receiving assistance with housing, transportation, older adult services, and federal benefits navigation. By addressing health-related social needs such as those that are often associated with increased health care use, CHW care can improve care at home after discharge. These findings align with CHW core competencies and emphasize the impact of CHWs in chronic disease management [39-41]. In some cases, participants also reported learning how to engage with service agencies independently, suggesting that the intervention promoted some skill-building in this area.

We found that although participants frequently credited CHW care with enhancing their clinical knowledge of HF through personalized health care coaching, few used the digital library of educational videos associated with the mobile app. These videos were expected to be a key source of asynchronous learning and skill-based reinforcement for patients. However, despite frequent use of other aspects of the digital platform (eg, digital blood pressure cuff, weight scale, and daily questionnaire), few participants watched the videos with regularity despite encouragement from CHWs. This highlights the challenge associated with how patients access aspects of digital interventions. In retrospect, the 3-item task list for daily completion of platform activities that patients used each day did not include the videos as an itemized task. Incorporation of an option to watch educational videos as a part of the task list may augment use if adopted in future mobile app iterations.

Finally, several participants identified technical challenges related to digital platform use. Prior to the performance of this study, we conducted an open pilot trial (single arm) specifically designed to optimize workflow integration and identification of any technical issues related to the digital platform [18]. Despite the identification and reconciliation of digital platform connectivity, syncing, capture, and accuracy issues in the prior open pilot trial, participants in this study experienced a number of technical challenges. Previous studies have shown that technical glitches in remote monitoring devices can lead to decreased patient adherence and trust [42,43]. In HF-focused digital platforms, the importance of reliable operability and connectivity cannot be overemphasized [44]. Ultimately, performance accuracy and precision will decide which digital platforms merit larger-scale integration in health systems.

### Limitations

This study has limitations. First, participants were recruited from a single large urban academic medical center, which may limit the generalizability of findings for patients in community or rural health systems. Second, because enrollment was limited to patients recently hospitalized for HF, the sample may reflect individuals with greater clinical and social complexity than the broader HF population. Although there are numerous studies examining digital platforms in HF cohorts [45,46] and examining CHW care in chronic disease or cardiovascular populations [47,48], the ability to discern specific effects of either the digital platform or CHW care alone within this study is limited. Although the study guide was comprehensive and thematic saturation was achieved, some patient perspectives may have still been underrepresented, particularly those of individuals who were less engaged. Finally, resource constraints limited trial enrollment eligibility to those with English proficiency, which may have excluded specific perceptions experienced by those patients fluent in languages other than English. We also acknowledge that insights from the control group were not included here and may have provided additional findings. Despite this, we believe that these limitations were balanced by a robust study design with authentic responses gathered from participants engaged in this study.

### Conclusions

This qualitative study demonstrated that patients with HF reported positive experiences with a digitally enabled CHW intervention. Most of the participants expressed that the components of the intervention worked well together and that the CHW care reduced barriers to platform use. These findings can inform digital and CHW home HF management for cardiac and primary care teams caring for this population. Further research is needed to better understand the facilitators and barriers to implementing this type of intervention in larger HF populations.

## Supplementary material

10.2196/93288 Multimedia Appendix 1 Patient interview guide.

10.2196/93288 Multimedia Appendix 2 Patient characteristics.

## Abbreviations

CHW: community health worker
HF: heart failure
REDCap: Research Electronic Data Capture
