# Supplementary material for: Patient Perceptions of a Digitally Enabled Community Health Worker Intervention: Qualitative Study Among Pilot Trial Participants
Source: JMIR Cardio. 2026 Jun 4;10:e93288. doi: 10.2196/93288 (PMC13235956; doi:10.2196/93288)
Supplement: Multimedia Appendix 1 [file cardio-v10-e93288-s001.docx]

Appendix 1: Interview Guide for Digitally-enabled CHW Intervention

Thank you very much for agreeing to this interview. This is going to be all about your experience in the last 30 days after leaving the hospital.  We will also ask you some questions about your life in general, and any barriers to your care.  We recognize that you might not have experience or knowledge about all the topics, in which case we will skip that question and move on to the next. We very much appreciate the opportunity to learn from you.

I will be taking notes during our conversation. Also, I would like to record the discussion to make certain I have accurately heard everything you said. The recording will only be available to the study team and will be destroyed at the end of the study.  Anything you say in the interview will be kept strictly confidential. May I have your permission to record? [IF NO, TURN OFF RECORDER]. Do you have any questions before I begin?

- Can you tell me in your own words what it’s been like to have this technology in your home and use it everyday for the last month or just use it for the last month? (I’d like to hear about good things and bad things.)

- - What could have made it better or what was good about the technology?
  - How satisfied were you with your interaction with the mobile app?
    - - Probe:
        - Heart rate
        - Oxygenation
        - Steps Taken
        - Weekly questionnaires
        - Monthly questionnaires
        - Videos
        - Blood pressure monitor
        - Weight scale
  - Is there any kind of help that we could provide that could have made that better?
  - Were there any times over the last month that it just didn’t work out for you and made you not want to use it at all?
  - What was difficult?
  - What would have made it better?
  - What are some things that seemed like they worked really well?
  - What did you like and was there anything that you didn’t like?
- Can you tell me in your own words what it’s been like to work with a community health worker for the last month? (I’d like to hear about good things and bad things.)

- - What could have made it better or what was good about working with the community health worker?
  - Is there any kind of help that we could provide that could have made this CHW partnership better?
  - Were there any times over the last month that it just didn’t work out for you and made you not want to work with your community health worker at all?
  - What was difficult?
  - What would have made it better?
  - What are some things that seemed like they worked really well?
  - What did you like and was there anything that you didn’t like?
- Overall how satisfied were you with the support you got from combined CHW and digital intervention?
- What could have improved the CHW and digital interactions for you?
- How likely would you be to use this type of CHW digital intervention again?
- How likely would you be to recommend this type of support to others that are living with heart conditions at home?
