# Supplementary material for: Patient Perceptions of a Digitally Enabled Community Health Worker Intervention: Qualitative Study Among Pilot Trial Participants
Source: JMIR Cardio. 2026 Jun 4;10:e93288. doi: 10.2196/93288 (PMC13235956; doi:10.2196/93288)
Supplement: Multimedia Appendix 2 [file cardio-v10-e93288-s002.docx]

Appendix 2. Participant Characteristics

| **Participant Characteristics** | **Intervention N= 19** |
| --- | --- |
| Female sex, N (%) | 9 (47) |
| Age, years, mean (SD) | 62.1 (15.1) |
| White, non-Hispanic, N (%) | 11 (58) |
| Black, non-Hispanic, N (%) | 3 (16) |
| Hispanic/ Latino , N (%) | 2 (11) |
| Asian, non-Hispanic, N (%) | 2 (11) |
| More than one race, N (%) | 1 (5) |
| Medicare, N (%) | 8 (42) |
| Medicaid/ MassHealth, N (%) | 1 (5) |
| Commercial/Private, N (%) | 9 (47) |
| Other, N (%) | 1 (5) |
| Ejection Fraction <40 %, N (%) | 10 (53) |
| ≤High school, N (%) | 6 (32) |
| Some college or ≥2-year college degree, N (%) | 13 (68) |
| Hypertension, N (%) | 14 (74) |
| Coronary Artery Disease, N (%) | 6 (32) |
| Diabetes, N (%) | 5 (26) |
| Hyperlipidemia, N (%) | 8 (42) |
| Arrhythmia , N (%) | 11 (58) |
| Chronic Kidney Disease, N (%) | 7 (37) |
| Depression/Anxiety, N (%) | 6 (32) |
| Hospitalizations in 12 months prior to enrollment, N (%) | 19 (100) |
| Indicated that they knew how to use a mobile phone or app for health purposes, N (%) | 17 (90) |
| Indicated that a digital platform would be able to help them achieve their goals for managing their heart condition at home, N (%) | 16 (84) |
